# Comprehensive microsurgical anatomy of the middle cranial fossa: Part I—Osseous and meningeal anatomy

**DOI:** 10.3389/fsurg.2023.1132774

**Published:** 2023-03-24

**Authors:** Ali Tayebi Meybodi, Giancarlo Mignucci-Jiménez, Michael T. Lawton, James K. Liu, Mark C. Preul, Hai Sun

**Affiliations:** ^1^ Department of Neurosurgery, Rutgers New Jersey Medical School, Newark, NJ, United States; ^2^ The Loyal and Edith Davis Neurosurgical Research Laboratory, Department of Neurosurgery, Barrow Neurological Institute, St. Joseph’s Hospital and Medical Center, Phoenix, AZ, United States; ^3^ Department of Neurosurgery, Barrow Neurological Institute, St. Joseph’s Hospital and Medical Center, Phoenix, AZ, United States; ^4^ Departments of Neurosurgery and Otolaryngology, Robert Wood Johnson Barnabas Health, Newark, NJ, United States; ^5^ Departments of Neurosurgery, Robert Wood Johnson Medical School, Rutgers University, New Brunswick, NJ, United States

**Keywords:** cavernous sinus, facial nerve, labyrinth, middle fossa, skull base dura, skull base foramina, sphenoid bone, temporal bone

## Abstract

The middle cranial fossa is one of the most complex regions in neurosurgery and otolaryngology—in fact, the practice of skull base surgery originated from the need to treat pathologies in this region. Additionally, great neurosurgeons of our present and past are remembered for their unique methods of treating diseases in the middle fossa. The following article reviews the surgical anatomy of the middle fossa. The review is divided into the anatomy of the bones, dura, vasculature, and nerves—in two parts. Emphasis is paid to their neurosurgical significance and applications in skull base surgery. Part I focuses on the bony and dural anatomy.

## Introduction

The middle cranial fossa (MCF) cradles the temporal lobe and borders the brainstem, sella turcica, and the cavernous sinus. Its floor includes critical neurovascular structures and separates the otic apparatus and infratemporal fossa from the intracranial space. The presence of multiple canals, foramina, and grooves as well as the complex meningeal folds in the region of MCF makes for a complex anatomy. Perfect knowledge of these intricate relationships is crucial for safe and efficient surgical exploration of the MCF. The purpose of this work is to provide a comprehensive anatomical review of the MCF from a neurosurgical perspective. Part 1 focuses on the osseous and meningeal anatomy with emphasis on surgical relevance.

## Bony anatomy

**Figure 1:**
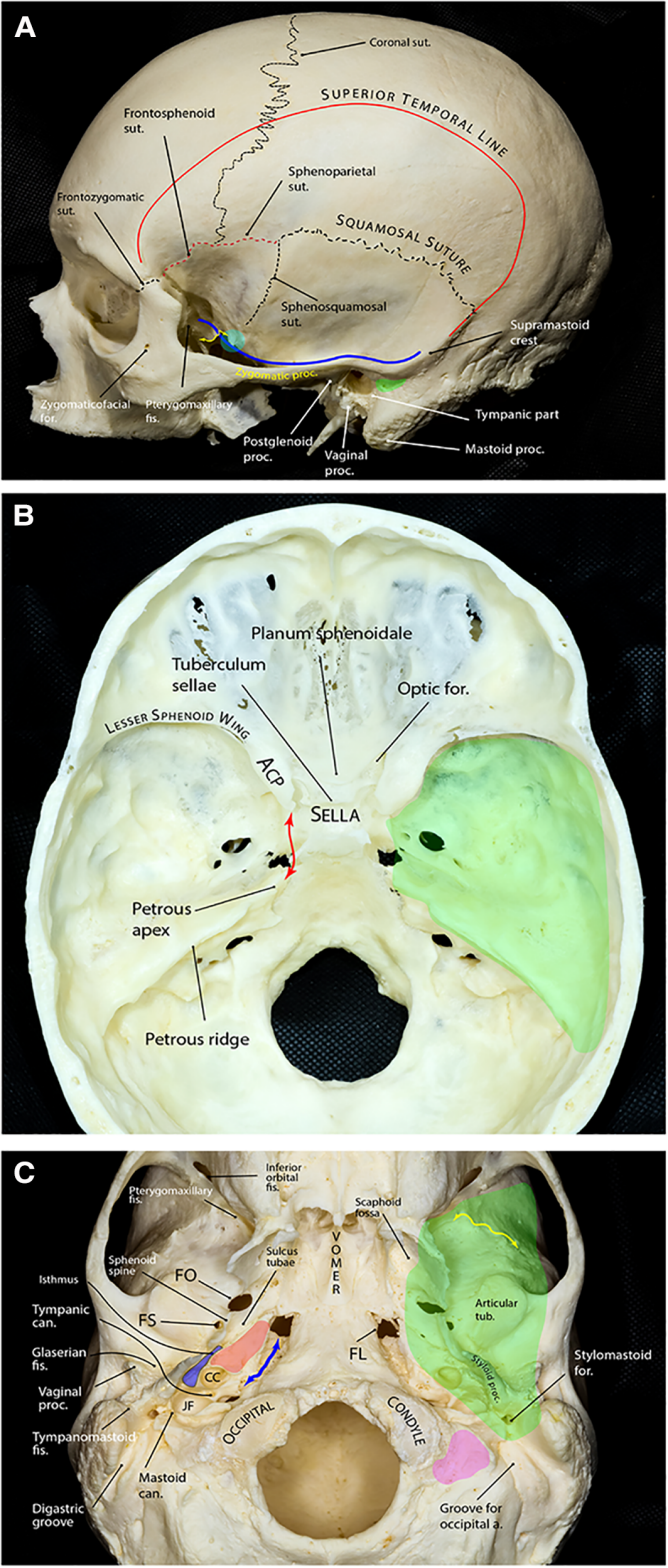
Bony anatomy of the middle fossa. (**A**) Lateral view of skull showing relevant osseous landmarks for middle fossa surgery. Superior temporal line is the line of attachment of the temporalis muscle. The greater sphenoid wing and the temporal squama slope laterally and superiorly from the middle cranial fossa. The blue line shows the contour of the middle fossa floor projected on the lateral skull surface. The floor is almost at the level of the zygomatic arch. The floor ascends posteriorly and is approximately at the level of the supramastoid crest, which continues as the superior temporal line. At the level of the supramastoid crest, the middle fossa is separated from the tympanic cavity by the tegmen tympani. The suprameatal (aka McEwen) triangle (green area) lies just inferior to the supramastoid crest and posterosuperior to the external auditory canal and is the gateway to the mastoid antrum during mastoidectomy. Further anteriorly, the middle fossa floor is composed of the greater wing of the sphenoid that makes the roof of the infratemporalfossa. A prominent rough bony tubercle (sphenoid tubercle, cyan circle), which serves as one of the attachment points of the deep temporal fascia, appears at the lateral end of the infratemporal crest (yellow double arrow) to which the lateral pterygoid muscle is attached. (**B**) Endocranial view showing the middle fossa proper (green area) separated from the sellar and parasellar compartments by the petrous-clinoid line (red double arrow). The posterior boundary of the middle fossa is formed by the petrous ridge. (**C**) Exocranial view of the bony skull base. The green shaded area shows the approximate projection of the middle fossa floor on the exocranial surface. The bony part of the pharyngotympanic tube (blue) runs parallel and lateral to the carotid canal (red area) with its anterior end turning into the cartilaginous portion at the region of sulcus tubae opening into the nasopharynx. Yellow double arrow marks the infratemporal crest and the petroclival fissure is marked by the blue double arrow. Pink area shows the jugular process of the occipital bone. a., artery; ACP, anterior clinoid process; can., canaliculus; CC, carotid canal; fis., fissure; FL, foramen lacerum; FO, foramen ovale; FS, foramen spinosum; for., foramen; JF, jugular foramen; proc., process; sut., suture; tub., tubercle. (Copyright Ali Tayebi Meybodi. Used with permission.)

The MCF is formed by two bones: sphenoid and temporal. Its floor is almost at the level of the zygomatic arch (Figure 1A). It extends from one side of the skull to the other with the sella turcica and upper clivus located in the middle, between the two cup-shaped spaces that house the temporal lobes of the brain. Therefore, the MCF can be divided into two distinct yet connected compartments: lateral and central. The lateral compartment contains the “middle fossa proper,” while the central compartment contains the sella turcica and the upper clivus. However, there is no obvious or distinct border between these two regions. An arbitrary line connecting the tip of the anterior clinoid process (ACP) to the petrous apex (i.e., petrous-clinoid line) may be considered as the transition line between the two compartments (Figure 1B).

### Boundaries

The middle fossa proper is delimited by the lesser sphenoid wing anteriorly, temporal squama, and the greater sphenoid wing laterally, petrous ridge posteriorly, and the petrous-clinoid line medially (Figure 1B). The lesser sphenoid wing is a slim lateral bony extension of the sphenoid body that forms the superior border of the superior orbital fissure (SOF). It gradually enlarges as it approaches medially and ends as the ACP. The lateral border of the middle fossa proper is formed mainly by the squamous part of the temporal bone (posteriorly) and the lateral upward extension of the greater sphenoid wing (anteriorly), which are separated by the sphenosquamosal suture. The posterior border is formed by the petrous ridge running from posterolateral to anteromedial. This ridge stops a few millimeters posterolateral to the lateral clival border. This space is occupied by the sphenopetroclival venous gulf and is home to Dorello's canal and posterior compartment of the cavernous sinus (1). Just posterior to the petrous apex, there is a gentle depression in the petrous ridge that houses the trigeminal nerve; hence, the structure is called the trigeminal depression. Marching posteriorly along the petrous ridge, the ridge forms a small ledge over the transverse-sigmoid junction and finally reaches the squamous part of the temporal bone at a point called the posterior petrous point (2).

Exocranially, the anterior MCF border is formed by the inferior orbital fissure (IOF) and a line that runs laterally along the anterior border of the infratemporal fossa just at the root of the temporal process of the zygoma. The medial border of the MCF starts as a line from the medial end of the pterygomaxillary fissure coursing posteriorly and crossing the pterygoid process of the sphenoid bone toward the foramen lacerum. It then continues further laterally on the lateral side of the petroclival fissure and medial to the exocranial orifice of the carotid canal and jugular foramen where it reaches its posterior point just lateral to the jugular process of the occipital bone and stylomastoid foramen (Figure 1C).

### Anterior clinoid process

**Figure 2:**
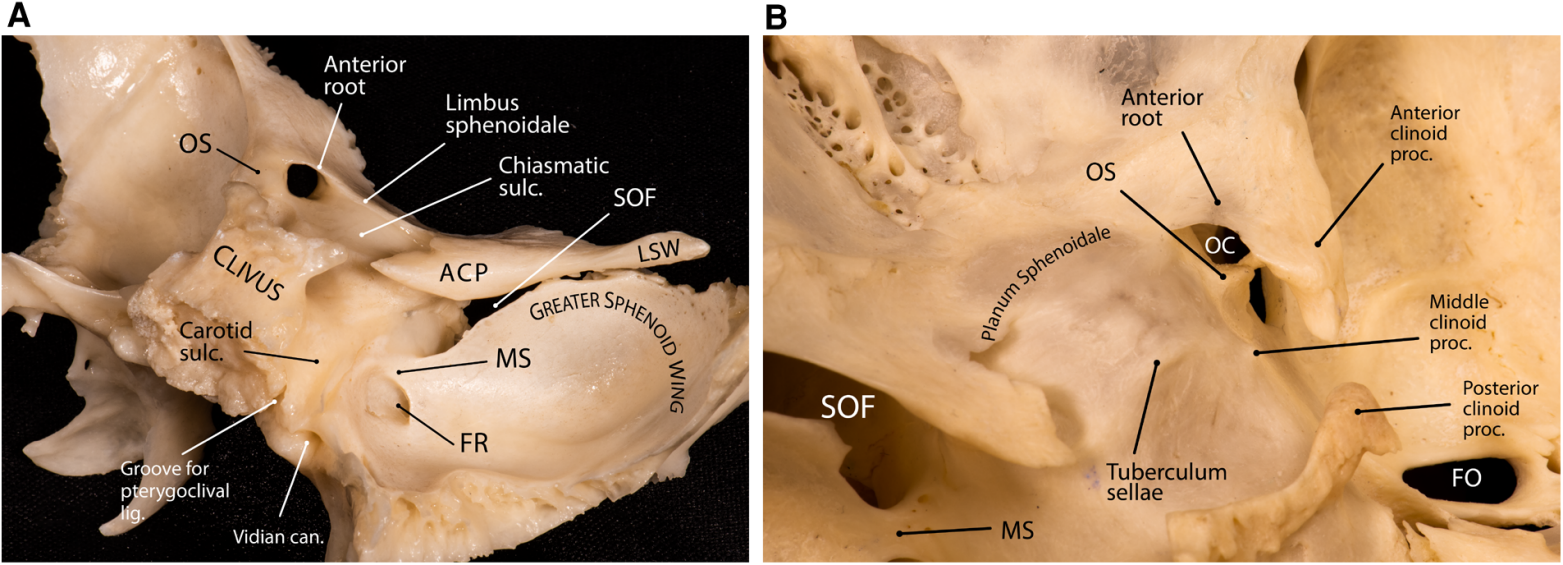
Anatomy of the anterior clinoid process. (**A**) Lateral posterior view of the sphenoid bone showing the relationship between the sphenoid wings, anterior clinoid and the sellar region. The superior orbital fissure is the cleft between the lesser and greater sphenoid wings. (**B**) Superior view of the central skull base and middle fossa. Note the interrelationship of the anterior clinoid roots. The tips of the anterior and middle clinoid processes partially encircle the internal carotid artery and may form a carotico-clinoid foramen around the carotid artery. ACP, anterior clinoid process; can., canal; FO, foramen ovale; FR, foramen rotundum; lig., ligament; LSW, lesser sphenoid wing; MS, maxillary strut; OC, optic canal; OS, optic strut; proc., process; SOF, superior orbital fissure; sulc., sulcus. (Copyright Ali Tayebi Meybodi. Used with permission.)

The ACP is a tooth-like medial extension of the lesser sphenoid wing that is attached to the body of the sphenoid bone by two “roots”: anterior and posterior (see below) (Figure 2). The tip of the ACP may form a bony bridge with the middle clinoid process creating an osseous ring around the internal carotid artery (ICA) across its transition from the clinoidal segment to the ophthalmic segment known as the carotico-clinoid foramen. The tip of the ACP may also be connected to the posterior clinoid process forming the interclinoid bridge. Both these osseous variants are relatively uncommon (3%–5% incidence in different studies).

### Optic strut

The posterior root of the ACP is called optic strut (OS)—also known as the “optic pillar” or “sphenoid strut” (3, 4). The OS is an important landmark during the removal of the ACP (i.e., anterior clinoidectomy) and has been colloquially named the “Rosetta Stone” of the paraclinoid region (5). This bony pillar connects the body of the sphenoid bone to the ACP and forms the floor of the optic canal while also separating the optic canal from the SOF. The mean dimensions are 6.54 ± 1.69 mm (length), 4.23 ± 0.69 mm (width), and 3.01 ± 0.79 mm (thickness) (6). It slants superolaterally toward the base of the ACP at an angle of about 40° from the vertical plane (3, 6). The point of attachment of the OS to the sphenoid body is variable relative to the chiasmatic sulcus (Figure 2A). According to Kerr et al., this point of insertion can be pre-sulcal (i.e., anterior or adjacent to the limbus sphenoidale) (12% incidence), sulcal (i.e., adjacent to the anterior two-thirds of the chiasmatic sulcus) (44% incidence), post-sulcal (i.e., posterior to the anterior two-thirds of the chiasmatic sulcus) (30% incidence), or asymmetric (14% incidence) (6). The sharp posterior margin of the OS is concave from side to side and accommodates the anterior surface of the ascending portion of ICA in the cavernous sinus, which essentially marks the superior end of the carotid sulcus at the lateral aspect of the sphenoid body.

**Figure 3:**
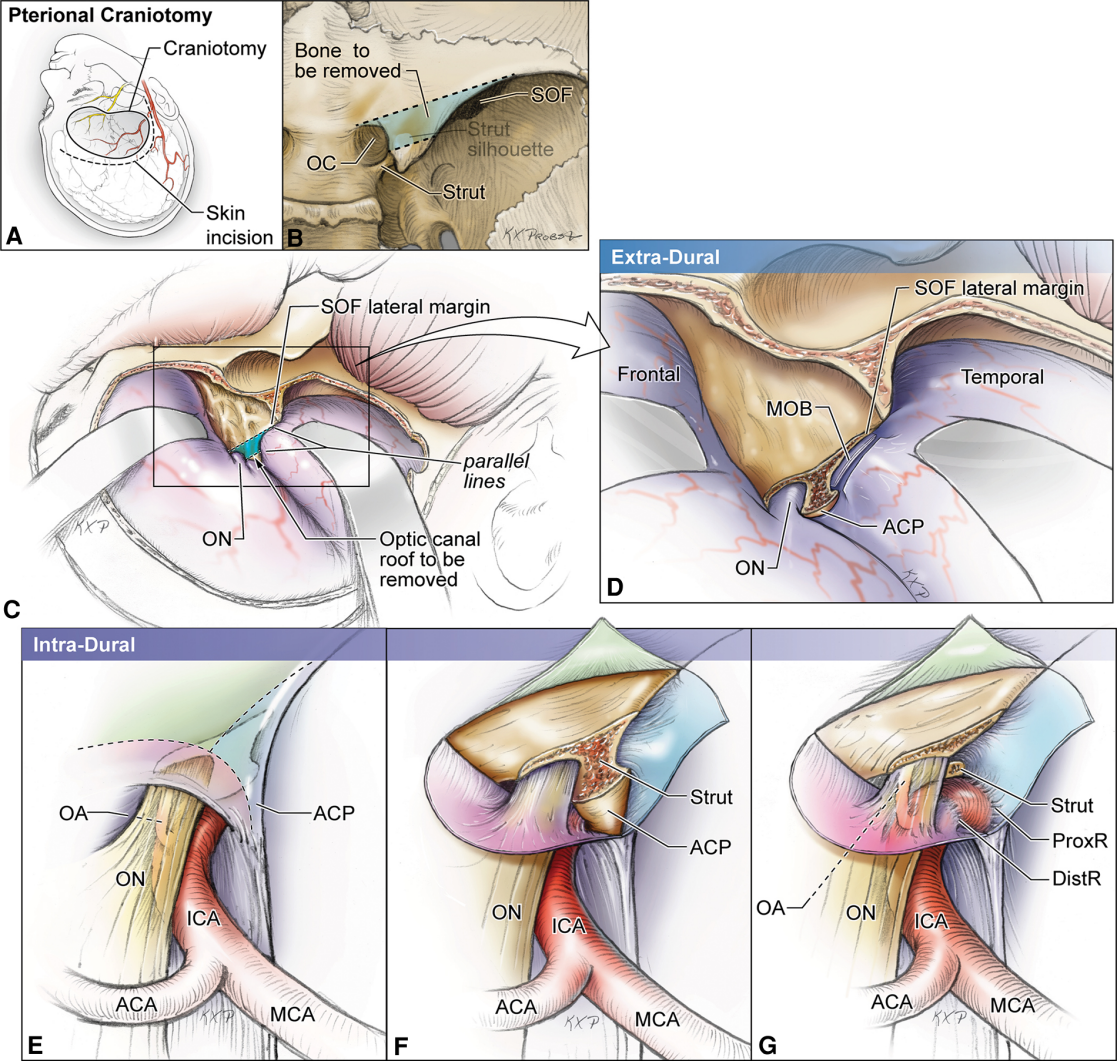
Artist's illustration of the two-step hybrid anterior clinoidectomy using skull base landmarks to identify the location of the optic strut. (**A,B**) Craniotomy and skin incision. Once the optic canal is identified extradurally, a line is drawn from its medial aspect to the lateral end of the superior orbital fissure. Next, a parallel line is drawn from the lateral aspect of the optic canal and the bony area between these lines delineates the anterior root of the anterior clinoid. (**C,D**) Extradural stage. The meningo-orbital band is cut, and the trapezoid area of bone between the two lines is drilled out and the strut is exposed. This stage unroofs the OC. (**E–G**) Intradural stage. Cuts are placed over the ACP dura to expose the tip of the clinoid process and the optic strut, which will be removed during this stage. ACA, anterior cerebral artery; DistR, distal dural ring; MCA, middle cerebral artery; MOB, meningo-orbital band; OA, ophthalmic artery; OC, optic canal; ON, optic nerve; ProxR, proximal dural ring; SOF, superior orbital fissure. (Copyright Michael T. Lawton. Used with permission.)

Understanding the location of the OS while drilling the ACP is essential to protect both the optic nerve and the ICA. The OS is usually at the level or anterior to a line drawn from the medial end of the SOF to the lateral corner of the optic canal (Figure 3) (7). Also, it is important to note that the ACP may be pneumatized when the aeration of the sphenoid sinus extends through the OS or the anterior root, which has an overall incidence of about 10% (8, 9). Assessment of the degree of pneumatization may help in the determination of the extent of an anterior clinoidectomy to avoid a plunge into the sphenoid sinus (10, 11) or, in general, to prepare for proper management of sphenoid sinus exposure (12).

### Anterior root of ACP

The anterior root is the medial extension of the ACP toward the planum sphenoidale. This sheet of bone is flat externally and concave internally, forming the roof of the optic canal. Its posterior margin blends with the limbus sphenoidale, which is a smooth crest making the border between the chiasmatic sulcus and the planum sphenoidale. During anterior clinoidectomy, this bony bridge needs to be drilled off (Figure 3). Care must be taken to avoid drilling the optic nerve as the anterior root may be quite thin.

### Floor

**Figure 4:**
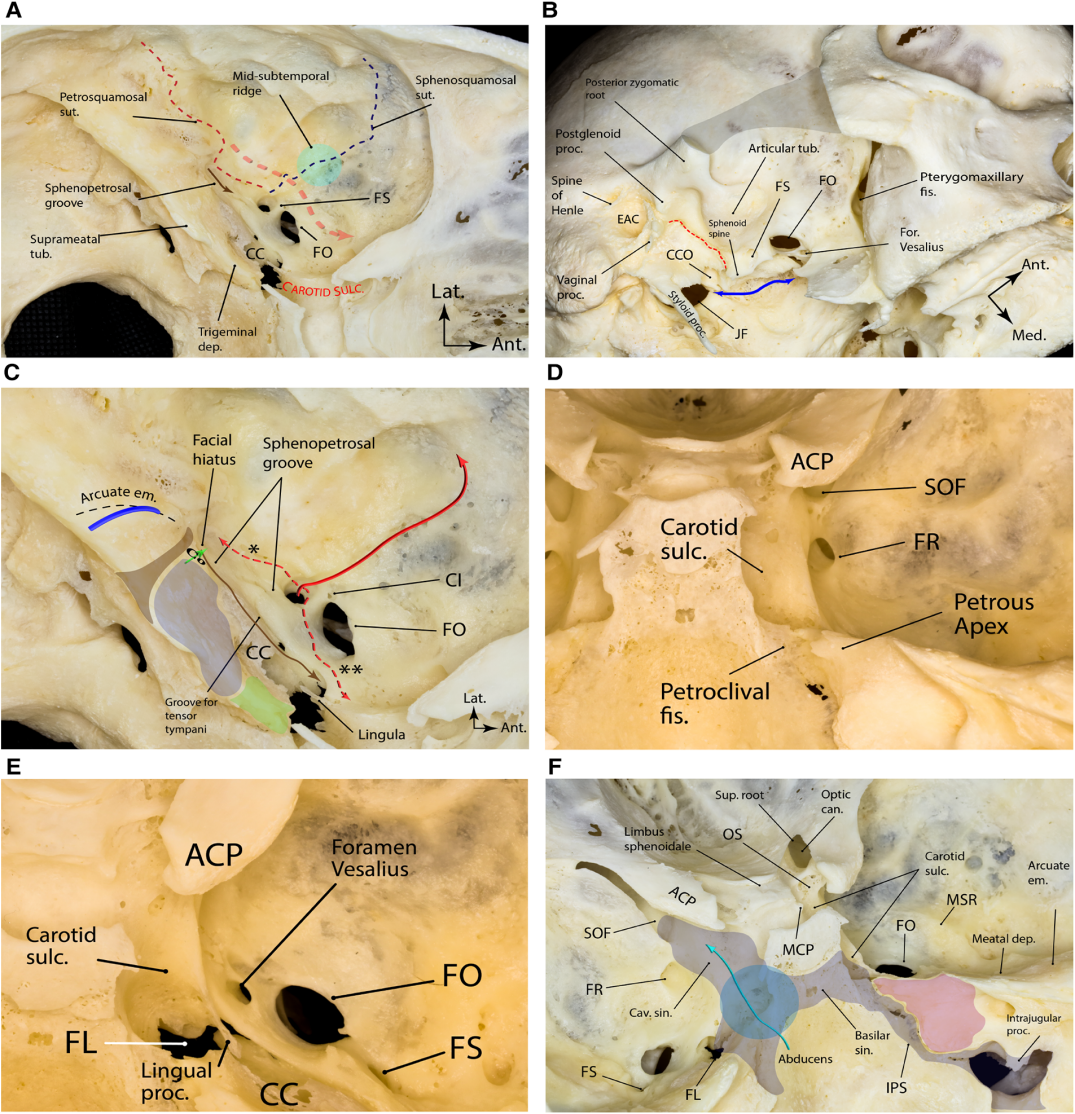
Bony landmarks of the middle fossa proper. (**A**) Note the two major bony sutures emanating from the lateral aspect of the foramen spinosum that harbors the lateral triangle of the middle cranial fossa, which is the corridor to exposing the pterygoid portion of the internal maxillary artery (red dashed arrow). The sphenopetrosal groove (brown arrow) is the pathway for the greater superficial petrosal nerve. (**B**) Oblique inferior view of the exocranial surface of the middle cranial fossa. The gray shaded area shows the resected zygomatic arch. The blue double arrow marks the exocranial petroclival fissure ending posteriorly in the jugular foramen. The sphenoid spine is found just medial to the foramen spinosum and is the point of attachment of the sphenomandibular ligament. The dashed red line marks the petrotympanic (Glaserian) fissure through which the chorda tympani nerve passes to join the lingual nerve. (**C**) Bony anatomy of the petrous apex. The brown shaded area shows the projection of the funnel-shaped internal auditory canal as the posterior boundary of the middle fossa rhomboid (stone blue shaded area) and the true petrous apex (green shaded area). Posterior to the internal auditory canal, one could find the arcuate eminence, which approximates the underlying superior semicircular canal (blue arch). Cochlea (Co) lies just anterior to the fundus of the internal auditory canal (green arrow showing the trajectory of the modulus axis). Tensor tympani muscle (brown arrow) lines the superomedial aspect of the osseous portion of the pharyngotympanic tube. The middle meningeal artery (solid red arrow) enters the skull through foramen spinosum and gives two important branches (dashed red arrows); the petrous branch (*) is the posterior, and the cavernous branch (**) is the anterior branch. (**D**) Posterior view of right middle fossa showing the relationship of the foramen rotundum with adjacent bony landmarks. (**E**) Superior view of right middle fossa showing the foramen Vesalius anteromedial to the foramen ovale. (**F**) Magnified endocranial view of the petrous face and its relationship with middle fossa and sellar bony anatomy. Note the relationship between the venous compartments of the posterior and middle fossae around the clivus. Also, note the region of sphenopetroclival venous gulf (blue circle) as a major confluence between the basilar, inferior petrosal, superior petrosal, and cavernous sinuses. The purple area is the petrous apex. Ant., anterior; can., canal; CC, carotid canal; CI, canaliculus innominatus; CCO, carotid canal orifice; cav., cavernous; dep., depression; EAC, external auditory canal; em., eminence; fis., fissure; for., foramen; FL, foramen lacerum; FO, foramen ovale; FR, foramen rotundum; FS, foramen spinosum; JF, jugular foramen; IPS, inferior petrosal sinus; Lat., lateral; MCP, middle clinoid process; Med., medial; MSR, midsubtemporal ridge; OS, optic strut; proc., process; sin., sinus; SOF, superior orbital fissure; sulc., sulcus; sup., superior; sut., suture; tub., tubercle. (Copyright Ali Tayebi Meybodi. Used with permission.)

The floor of the middle fossa proper is formed by the superior surface of the petrous pyramid posteriorly and the greater wing of the sphenoid anteriorly. The greater wing is concave and harbors all foramina of the middle fossa. On the other hand, the petrous surface is convex. Two distinct bony sutures can be identified here, both emanating from the lateral aspect of the foramen spinosum (FS): the petrosquamosal and sphenosquamosal sutures, which form an angle of roughly 90° with each other (Figure 4A). Several foramina and bony protuberances are found on the middle fossa floor, which have neurosurgical implications, as follows.

### Middle fossa foramina

#### Foramen spinosum

First described by Jakob Benignus Winslow (1669–1760) in the 18th century, FS is the posteriormost and lateralmost foramen in the middle fossa floor with an average diameter of 1 mm (0.5–2.0 mm) (Figure 4A) (13). The FS is named after a small spinous process found posterior to it on the exocranial surface of the skull known as the sphenoid spine (Figure 4B). The FS harbors the middle meningeal artery (MMA), a venous plexus connected to a plexus around the V3 division of the trigeminal nerve at foramen ovale (FO), the cavernous sinus endocranially and the pterygoid venous plexus exocranially, and a recurrent meningeal branch of the V3 (i.e., nervus spinosus). On average, the FS is 5 mm posterolateral to foramen ovale (range 2.0–7.5 mm) (13) and 25 mm (range, 17.8–33.1) medial to the lateral border of the middle fossa (2). FS may sometimes be duplicated (14).

#### Foramen ovale

Almost 5 mm anteromedial to FS, there is an oval foramen harboring the mandibular branch of the trigeminal nerve (V3), accessory middle meningeal artery (in case it exists), an emissary vein connecting the adjacent cavernous sinus to the exocranial pterygoid plexus, and the lesser petrosal nerve (LPN). The FO opens into the underlying infratemporal fossa (Figures 4A,B). FO can vary in shape, from completely round to almond-shaped or slit-like (14). It has an average size of 7 × 4 mm (15) and is, on average, located 30 mm (range, 24.4–39.8) from the lateral border of the middle fossa (2).

#### Canaliculus innominatus

Canaliculus innominatus (of Arnold) (CI)—also known as the foramen petrosum or foramen of Arnold—is a small foramen that is occasionally found between the FS and FO that acts as a conduit for the LPN (Figure 4C) (16–18). It has an incidence of 17%. Kakizawa et al. found that most LPNs pass through the CI to exit the middle skull base. Specifically, they found that LPN crossed the middle fossa floor anterior to the greater petrosal nerve and exited the middle fossa through the CI in 70% (14/20) of cases where a CI was present (19).

#### Foramen rotundum

The foramen rotundum (FR) is the most anterior and medial foramen of the middle fossa. It is oriented rather vertically than horizontally, unlike the orientation of both the FS and FO (Figure 4D). The FR's reported dimensions are 4 × 3 mm, located about 8–10 mm anteromedial to FO (20). The FR transmits the V2 division of the trigeminal nerve. It is also located inferior to the medial end of SOF and separated by a thin bony bridge called the maxillary strut. The maxillary strut is an important landmark in endoscopic endonasal approach to the MCF and lateral wall of the cavernous sinus (21).

#### Foramen Vesalius

The foramen Vesalius (FV), also known as the emissary sphenoidal foramen, is a small, variable but consistently symmetrical foramen located anteromedial to the FO and lateral to the FR (Figures 4B,E) (22, 23). When present, it contains an emissary vein connecting the pterygoid venous plexus in the infratemporal fossa with the cavernous sinus in the middle fossa. The prevalence of FV varies from study to study, reported between 5% and 60% (22). It is an important structure because it can be a potential channel for transmitting sepsis from extracranial veins to intracranial venous sinuses. In addition, when treating trigeminal neuralgia with trigeminal rhizotomy through the trans-ovale approach, the needle can be misplaced in the FV and puncture the cavernous sinus, causing severe intracranial bleeding (22, 24). During middle fossa approaches, care must be taken to identify this anatomic variation in preoperative images and not to mistake it for FO or FR.

#### Superior orbital fissure

The SOF is a narrow bony cleft through which the orbit communicates with the middle cranial fossa—situated between the greater and lesser wings and body of the sphenoid bone (Figures 2A, 4F) (25). The superior wall of the fissure is formed by the lower surfaces of the lesser wing, the ACP, and the adjacent part of the OS. The fissure allows the passage of many structures, such as the oculomotor, trochlear, ophthalmic, and abducens nerves, branches of the carotid sympathetic plexus, as well as superior and inferior ophthalmic veins. Part of the annular tendon (i.e., annulus of Zinn) from which the rectus muscles arise is attached to the bony boundaries of the SOF and crosses the fissure. Infrequently, the anomalous ophthalmic artery may pass through the SOF. The SOF is exposed during the anterolateral approaches to the central skull base and lateral sellar compartment (26).

### Middle fossa canals and grooves

#### Facial hiatus and the sphenopetrosal groove

Medial to FS and almost parallel to the petrous ridge, there is a shallow and sometimes inconspicuous groove called the sphenopetrosal groove, which harbors the greater superficial petrosal nerve (GSPN)—the GSPN starts from a small bony opening posteriorly known as the facial hiatus (i.e., hiatus falopii) and ends underneath the V3 (Figure 4A). In its complete form (10% of middle fossae), this sphenopetrosal groove (or simply GSPN groove) ends at the posterior rim of the V3 about 7.5 ± 2.9 mm (range 3.0–12.0) posterior to the FO (27, 28). However, in the majority of cases (65%), the groove is not complete and is absent in 25% of middle fossae (28). The posterior end of the groove that usually corresponds to the facial hiatus is about 25 mm (range 14.7–33.1) medial to the lateral border of the middle fossa in the coronal plane (i.e., external surface of the squamous temporal bone) (2). The length of the sphenopetrosal groove is on average 10–12 mm (range, 6.0–15.0) (27, 28). It is located on top of the petrous ICA although the exact relationship is variable), separated from by a thin shell of bone. In 15% of cases, the bone is dehiscent (29).

The facial hiatus is the bony opening where the nervus intermedius fibers originating in the superior salivatory nucleus of the brainstem exit the geniculate ganglion of the facial nerve to continue as the GSPN en route to the pterygopalatine ganglion. The size of the facial hiatus may vary depending on the degree of ossification of the middle fossa on top of the geniculate ganglion. This bony coverage is incomplete or totally absent in 15%–25% of specimens (28, 30).

#### Groove for the middle meningeal artery

The MMA carves a conspicuous groove on the middle fossa floor after exiting FS. This groove is directed anteriorly on the greater sphenoid wing toward the lesser sphenoid wing and then ascends laterally as it approaches the pterion (Figure 4C). The posterior division of the MMA branches from this middle fossa segment variably along its length and courses laterally and posteriorly.

### Middle fossa protuberances

#### Midsubtemporal ridge/tubercle

The midsubtemporal ridge/tubercle (MSR) is a boomerang shaped peak in the region of sphenosquamosal suture found in 90% of middle fossae described by Wanibuchi et al. (Figure 4F) (31). On average, it is 3 mm in height, 6 mm wide, and 9 mm long. The authors reported the distance between the midpoint of MSR and the midpoint of a line connecting FO and FR to be 11 mm on average. This bony protuberance is lateral to the FS and should not be mistaken with the arcuate eminence (AE), which is subtler and located posteromedial to the FS (see below). The MSR is a useful landmark to localize the interval between the V2 and V3 [i.e., the anterolateral triangle (see Part 2)].

#### Arcuate eminence

The superior semicircular canal (SSC) was introduced by Fisch as a consistent landmark for finding the internal auditory canal (IAC) during middle fossa surgery (32). He mentioned that the location of the SSC can be consistently determined by the AE that overlies it (32). The AE is a bony protuberance anterior to the tegmen tympani and posterior to the IAC and geniculate ganglion. Current opinion is that the existence of AE is the result of combined effect of SSC, pneumatization of the petrous bone and the occipitotemporal sulcus of the basal surface of the temporal lobe (33). AE is conspicuous in 85% of middle fossae and almost absent in the rest (33, 34). It may assume different shapes from a single linear arc to double-arc or complex geometric morphology. It is directed from posteromedial to anterolateral. In about 95% of cases, the axes of SSC and AE subtend an angle <45° (33). Kartush et al. found that the relationship between the SSC and AE is consistent but not exact, i.e., the lateral point of the AE almost always overlies the lateral limb of the SSC; however, the medial point of AE usually deviates posteriorly (never anteriorly) compared to the medial limb of SSC (Figure 4C) (34). The dome of AE is usually 22 mm (range, 18–25) medial to the inner table of the skull in the coronal plane (2, 35). It is almost always found posterior to the coronal level of the posterior root of the zygoma (Figure 1A) (36). On average, the AE dome is 4.2 mm superior, 5.3 mm lateral, and 6.3 mm posterior to the dome of the corresponding SSC (35).

### Deep anatomy

#### Carotid canal

**Figure 5:**
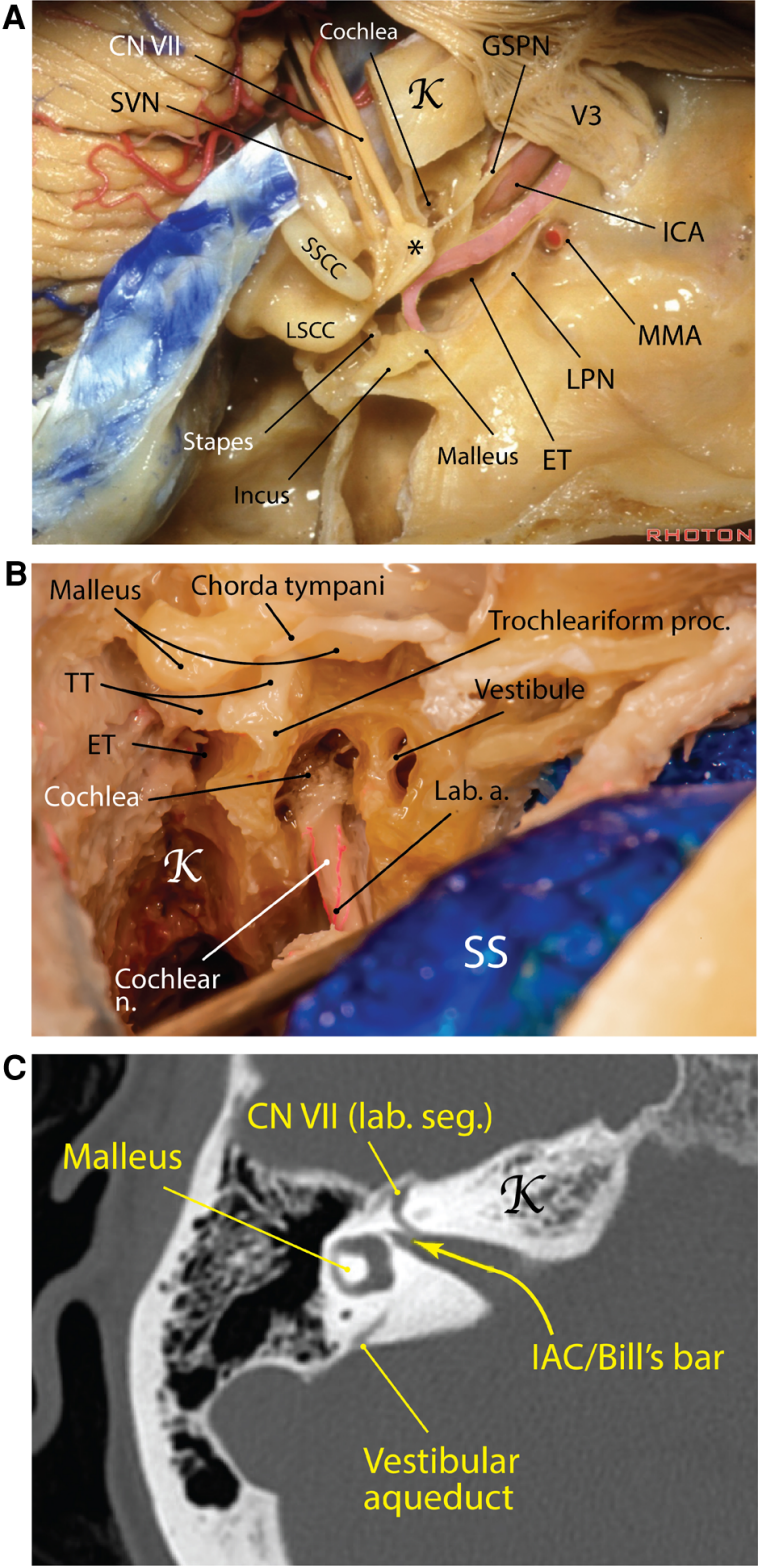
Carotid canal Eustachian tube in the middle cranial fossa. (**A**) The carotid canal starts at the exocranial surface of the temporal bone anteromedial to the jugular foramen. The GSPN runs on top of the petrous ICA and a thin shell of bone may separate the two. The Eustachian tube runs parallel and lateral to the carotid canal and harbors the tensor tympani muscle (pink area). *Geniculate ganglion [Courtesy of *The Rhoton Collection*, American Association of Neurological Surgeons (AANS)/Neurosurgical Research and Education Foundation (NREF)]. (**B**) A right mastoidectomy exposing the middle ear structures and the posterior end of the Eustachian tube, which is adjacent to the trochleariform process (Courtesy of Barrow Neurological Institute. Used with permission). (**C**) Right temporal bone computed tomography showing the internal auditory canal and the vertical crest (aka Bill's bar). a., artery; CN, cranial nerve; ET, Eustachian tube; GSPN, greater superficial petrosal nerve; ICA, internal carotid artery; K, Kawase's rhomboid; lab., labyrinthine; LPN, lesser petrosal nerve; LSCC, lateral semicircular canal; MMA, middle meningeal artery; n., nerve; proc., process; seg., segment; SS, sigmoid sinus; SSCC, superior semicircular canal; SVN, superior vestibular nerve; TT, tensor tympani.

The carotid canal is the longest and largest bony conduit in the human body. It is located in the petrous temporal bone, under the floor of MCF (Figures 1C, 4). As the name implies, it harbors the petrous segment of the ICA. However, it is also home to a thin pericarotid venous plexus as well as the carotid perivascular neural plexus and its major condensations (e.g., carotid nerve and deep petrosal nerve). The exocranial carotid foramen marks the entry of the ICA into the petrous bone and is located medial to the vaginal process of the tympanic bone and anterior to the jugular foramen (Figure 4B). The carotid canal conforms to the course of the petrous carotid and, therefore, has posterior vertical, posterior genu, and horizontal segments. The ICA exits the carotid canal just proximal to its anterior genu. As such, the anterior genu and anterior vertical segments of ICA are not housed in the carotid canal. The exocranial orifice of the carotid canal is located anterior to the jugular foramen, posterior to the Eustachian canal (junction of its osseous and cartilaginous parts), and medial to the tympanic part of the temporal bone and temporomandibular joint (Figure 4). The petroclival fissure ends at the anteromedial corner of the jugular foramen, medial to the carotid canal's exocranial orifice. The carotid canal courses anteromedially and ends at the posterolateral aspect of the foramen lacerum. The length of the carotid canal is 16–20 mm (37). The thickness of the bone of middle fossa floor on top of the carotid canal is variable, but it generally decreases as one marches along the carotid anteriorly. Notably, the middle fossa floor might be dehiscent above the carotid artery (Figure 5). Thus, assessment of preoperative CT images is critical in determining the individual variations to protect the ICA during drilling of Kawase and/or Glasscock triangles (see Part 2).

#### Eustachian tube

The Eustachian tube (ET), also known as the pharyngotympanic tube or the auditory tube, is named after Italian anatomist, Bartolomeo Eustachi (c. 1500–1510–1574). He was the first to observe a canal that connected the nasopharynx to the tympanic cavity of the middle ear. The ET has a role in equalization, oxygenation, and drainage of the tympanic cavity—specifically, it permits equalization of pressure in the middle ear with respect to ambient pressure (38). By doing this, it influences the tension exerted on the tympanic membrane and the attached ossicles, which indirectly affects the transmission of sound waves.

Starting from the tympanic ostium at the anteroinferior end of the tympanic cavity, the ET runs immediately parallel and lateral to the carotid canal and is usually separated from it by a thin bony shell of about 2 mm thickness (Figures 1C, 5) (39–41). The ET comprises two anatomically distinct parts: an osseous posterolateral and a fibrocartilaginous anteromedial portion. The bony canal is located lateral to the posterior genu of ICA in petrous canal and is usually comprised of two semicanals, one for ET proper and the other for the tensor tympani muscle, which is superomedial to the former (41). At the anterior orifice of the bony ET on the exocranial surface which lies just medial to the FS, a distinct bony sulcus (*sulcus tubae*) is extended anteromedially toward the petrous apex, housing the cartilaginous ET. The transition of bony ET canal to sulcus tubae is called the isthmus (narrowest part of ET) and is marked by the sphenoid spine located laterally at the level of FS (Figure 1C). The scaphoid fossa of the sphenoid bone is the portion of the exocranial bone harboring the most distal segment of the cartilaginous ET before joining the nasopharynx. The length of the ET ranges between 31 and 44 mm. Surgically, the cartilaginous ET may be further subdivided into 4 segments from posterior to anterior: petrous [adjacent to petrous (i.e., horizontal) ICA], lacerum (adjacent to lacerum ICA), pterygoid and nasopharyngeal (41).

Marching from posterior to anterior, the ET assumes a slightly downward and lateral trajectory toward the petrous apex to end in the lateral corner of the nasopharynx. Therefore, its nasopharyngeal opening is situated inferior to the anterior end of the petrous ICA. The anterior and of sulcus tubae lies lateral to the foramen lacerum and medial to the scaphoid fossa.

The tensor tympani muscle is attached to the length of the cartilaginous part of the ET and courses posteriorly to sharply turn around the trochleariform process (a thin bony prominence in the anterior middle ear cavity), where it transitions into a tendon, attaching to the handle of malleus. Most often (72%), the tensor tympani muscle is superior to the ET but it also could be anterior or posterior to it (42). Tensor tympani is usually covered by a thin bony shell on the MCF but there may be partial dehiscence (39). Drilling of the MCF lateral to the carotid canal (Glasscock's triangle) and mobilizing the ET could increase the exposure of the petrous carotid if needed (43).

#### Cochlea

The cochlea is the heart of the peripheral auditory apparatus. It is the most anterior and medial part of the labyrinth (Figure 5). The cochlea, located in the depth of the temporal bone, is formed as a spiral modulus similar to a snail shell with 2.5 turns. When unwound, it is almost 35 mm in length (44). The cochlear spiral is about 5 mm tall and the width of its base is about 9 mm. The modular axis and apex of the cochlea are directed inferolaterally with its base bulging into the anterior, inferior, and medial corner of the middle ear cavity as the promontory. The modular axis of the cochlea makes average angles of 60°, 25°, and 8° with the coronal, sagittal, and axial planes, respectively (45). Also, the modular axis is perpendicular to the petrous ridge. The basal turn is almost parallel to the GSPN and subtends an angle of 60° with the IAC on average. Immediately posterior and hidden under the niche of the basal turn of the cochlea in the middle ear cavity (promontory) are found the oval window (superiorly) and round window (inferiorly). The middle ear ossicles, vestibule, semicircular canals, and facial nerve all lie posterior to the cochlea. The cochlea is in close relationship with the labyrinthine and tympanic segments of the facial nerve as well as with the geniculate ganglion. This proximity is maximum between the basal turn and the labyrinthine segment (only 0.4 mm distance) (45). Cochlea has a special relationship with the labyrinthine segment of the facial nerve. Upon leaving the fundus of IAC, the facial nerve makes an anterior and medial turn above the basal turn of the cochlea to end in the geniculate ganglion (46, 47). The average distance between the labyrinthine segment of the facial nerve and the basal turn of the cochlea is 4.3 mm (45).

The trochleariform process is a tiny bony prominence on the superior aspect of promontory and anterior to the oval window and inferior to the tympanic segment of the facial nerve where the tendon of tensor tympani attaches (see above—“Eustachian tube”) (Figure 5). A delicate neural meshwork covers the promontory. This neural meshwork emanates from the tympanic branch of the glossopharyngeal nerve (aka Jacobson's nerve), which enters the middle ear cavity from inferiorly (i.e., jugular fossa) and finally unites to form the lesser petrosal nerve.

The cochlea lies slightly posterolateral and superior to the posterior genu of the IAC in the MCF. The closest distance between cochlea and the ICA is between the basal cochlear turn and the posterior ICA genu (average 1.9 mm) (45). This bone may be dehiscent (48). Marching posterolateral on the MCF from the cochlea is located the semicircular canals. The only prominence in the MCF harboring the semicircular canals is the AE (see above—“Arcuate eminence” section).

“Cochlear angle” is a term used to describe a bony region between the IAC and labyrinthine segment of the facial nerve and the GSPN. This bony region houses the cochlea and is an important landmark in MCF surgery. Basically, the basal and middle turns lie inferior to the labyrinthine segment of the facial nerve and the apical turn is situated inferior to the geniculate ganglion. The basal turn lies on average 4 mm below the MCF.

#### Intraoperative landmarks for cochlear protection

Approaches through the MCF can result in hearing loss due to cochlear damage. The cochlea is located anterior to the fundus of the IAC, in the angle between the IAC and GSPN, inferomedial to the geniculate ganglion. The part of the cochlea most frequently damaged is the basal turn. Utilizing the anterior petrosal approach, Kim et al. were the first to describe an anatomic line based on landmarks—called the cochlear line (CL)—to identify the cochlea and preserve hearing function (49). The CL cochlear line is a line drawn from the crossing point between the GSPN and the petrous ICA perpendicular to the line drawn over the apex of the superior circumference of the dura of the IAC. The CL marks the anteromedial perimeter of the cochlea. Using this line as a landmark resulted in a safety margin of approximately 2 mm (reported 2.25 mm) around the cochlear cavity, which does not inhibit the view of the surgical trajectory to the brainstem. However, using the CL is challenging due to positioning and potentially dangerous due to the exposure of neurovascular structures (e.g., petrous ICA).

In 2019, Guo et al. introduced another anatomical line for preserving hearing, called the cochlear safety line (CSL), utilizing the same anterior petrosal approach (50). The CSL projects from the lateral edge of the foramen ovale that intersects perpendicularly the upper dural transitional fold, i.e., a line between the roof and anterior wall of the IAC. Authors found that this line never intersected the cochlea, with an average distance of 1.82 mm ± 0.99 between the CSL to the bony cochlea and 2.78 mm ± 1.29 to the membranous cochlea.

#### Internal auditory canal

Between the trigeminal prominence (which is a subtle bump posterior to the trigeminal depression) and the AE on the MCF is a subtle depression called the meatal depression (Figure 4). The IAC lies roughly inferior to the meatal depression. The IAC is usually a funnel-shaped canal, which is wider medially, although it may be cylindrical or bud-shaped in a minority of specimens. Its medial porus sizes are 8.6 mm × 5.6 mm (anteroposterior × vertical), whereas at the fundus these dimensions are 2.5 mm × 4.0 mm (51). The porus lies about 5 mm below the level of the petrous ridge and 5 mm posterior to the posterior lip of the trigeminal depression. Marching laterally along the IAC, the thickness of MCF bone overlying the IAC becomes smaller—submillimeter above the labyrinthine segment of the facial nerve. This anatomic fact is very important to protect the contents of the IAC during drilling of the middle fossa. The basal turn of the cochlea is related to the anteroinferior aspect of the lateral end of the IAC. The fundus of IAC is the point where the facial and vestibulocochlear nerves exit the IAC harboring a transverse crest, which divides the fundus into superior and inferior compartments. The superior compartment is further divided by a subtle vertical crest also called “Bill's bar” in recognition of William House (Figure 5C) (52).

## Dural anatomy

As the temporal lobe sits in the cradle of the middle fossa, it is covered by the dura mater. Regarding its layered structure, the dura mater of the middle fossa proper is relatively simple laterally; i.e., it is composed of two adherent layers: meningeal and endosteal. However, as one marches from lateral to medial, this layered structure becomes more complex. First, it should be noted that all the foramina of the skull base are lined with the endosteal layer of the dura. Therefore, when the dura is detached from lateral to medial on the middle fossa floor, once one reaches a foramen (e.g., FS or FO), the endosteal layer of the dura must be incised for further dissection to be feasible.

### Dural folds of the anterior clinoid region

The dura over the ACP extends anteriorly, posteriorly, medially, and inferiorly. Anterior continuation of the ACP dura will lead to the anterior cranial fossa on the orbital roof. Posteriorly, it continues as the anterior petroclinoid and interclinoid ligaments connecting the ACP to the petrous apex and posterior clinoid process, respectively. Medially, the dura on the superior surface of the ACP merges with the dura of the planum sphenoidale after crossing the ICA (see below) and lines the margins of the intracranial ostium of the optic canal and continues around the optic nerve up to the sclera. The dural fold lining the inferior part of the ostium of the optic canal covers the upper surface of the optic strut. The ophthalmic artery usually originates from the first few millimeters of the intradural ICA. It runs between the optic nerve and the dura lining the superior part of the optic canal and continues interdurally (not intradurally) toward the orbit (53).

**Figure 6:**
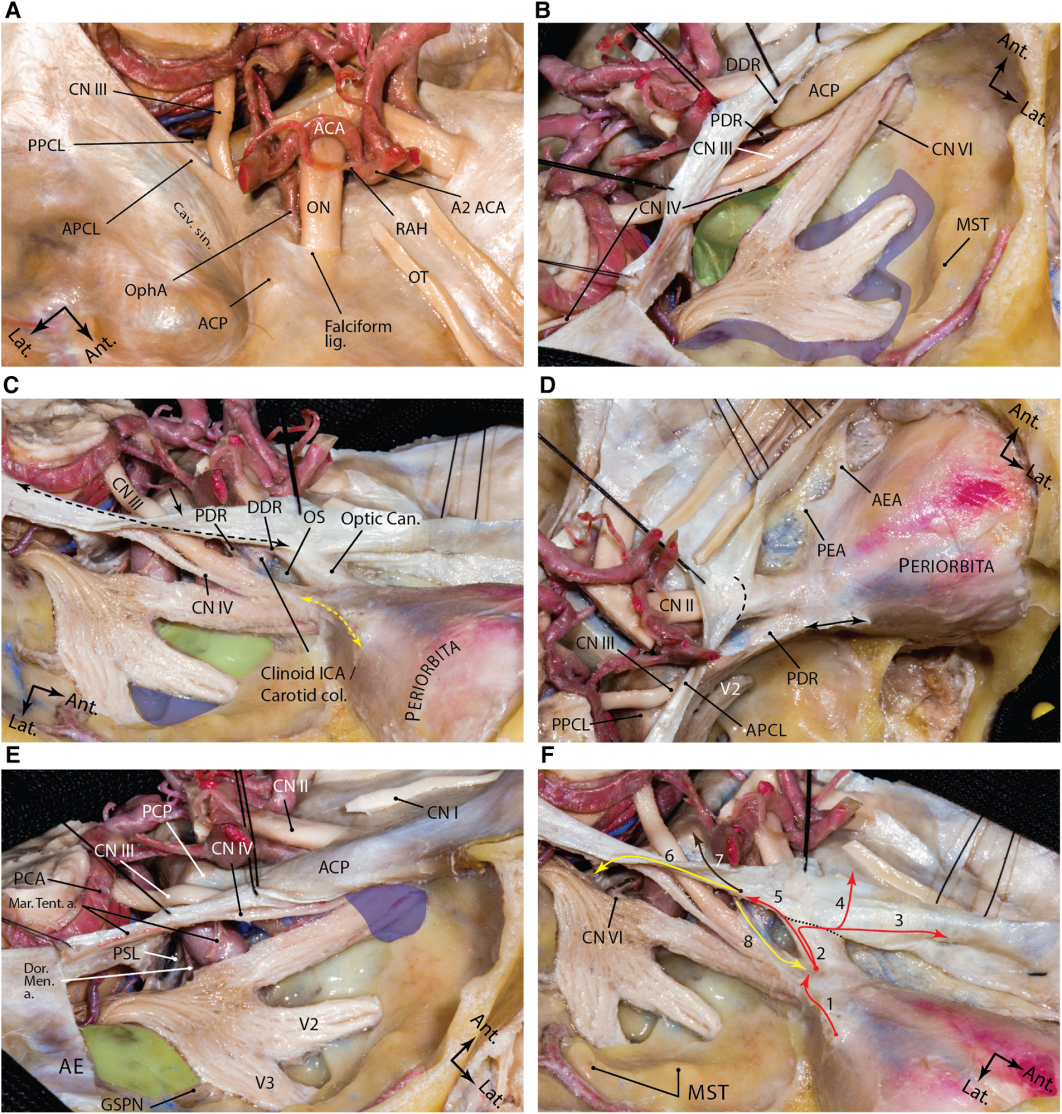
Dural anatomy of the middle fossa. (**A**) exposure of the middle fossa dura and its relationship with the dura and anatomic structures of the central skull base. (**B**) The meningeal and endosteal layers of the dural covering of the middle fossa are removed to expose the relevant neural relationships. Lifting the dura over the anterior clinoid process and along the lateral wall of the cavernous sinus. The proximal and distal dural rings are dural layers surrounding encompassing the anterior clinoid process. One may appreciate how trochlear nerve pierces the medial tentorial edge and enters the roof of the cavernous sinus. Meckel's cave orifice which is composed of meningeal layer of dura is widened around the trigeminal nerve root. This layer continues anteriorly as the roof of the cavernous sinus under the anterior clinoid process as the proximal dural ring. The green shaded area shows the Parkinson's triangle (the largest triangle of the cavernous sinus) through which the posterior genu of the cavernous carotid is exposed. The blue shaded area marks the laterotrigeminal venous system region. (**C**) Following anterior clinoidectomy, the relationship between the proximal and distal dural rings is better revealed. These layers converge at the root of the anterior clinoid process and are opened and continue laterally as the meningo-orbital band (dashed yellow double arrow) and anteriorly as the periorbita. Note the reflection of the meningeal layer of the dura (black arrow) along the tentorial edge (dashed black double arrow). Green and blue areas show and anteromedial and anterolateral triangles, respectively. (**D**) Upper view of the right cavernous sinus exposure and dural covering of the orbit along the cribriform plate. Black double arrow shows the amputated meningo-orbital band. Dashed line shows the location of the falciform ligament. (**E**) Exposure of the posterior aspect of the cavernous sinus and dural reflections around Meckel's cave entrance. Blue area shows the superior orbital fissure and green areas shows the middle fossa rhomboid (Kawase's). (**F**) Dural reflections in the paraclinoid region. 1, meningo-orbital band; 2, tip of the anterior clinoid process; 3, anterior fossa along the cribriform plate; 4, anterior fossa along the planum sphenoidale; 5, distal dural ring; 6, medial tentorial edge, anterior petroclinoid and petrosphenoid ligaments; 7, interclinoid ligament; 8, proximal dural ring. Black dashed line represents the location of falciform ligament. ACA, anterior cerebral artery; ACP, anterior clinoid process; ant., anterior; APCL, anterior petroclinoid ligament; cav., cavernous; CN, cranial nerve; lat., lateral; lig., ligament; ON, optic nerve; OphA, ophthalmic artery; OT, olfactory tract; PPCL, posterior petroclinoid ligament; RAH, recurrent artery of Heubner; sin., sinus. (*Courtesy of Barrow Neurological Institute. Used with permission*.)

The medial continuation of the dura on the superior surface of ACP encircles the ICA laterally along a line of attachment that is inclined superiorly from posteriorly to anteriorly, known as the distal dural ring (DDR) (Figure 6) (54, 55). The DDR encircles the ICA and merges anteriorly with the dural fold on the superior surface of the optic strut, which separates the ICA and the optic nerve at the posterior end of the optic canal (54).

The DDR attachment to the posteromedial wall of the ICA lies at a lower level than at the lateral wall of the ICA; thus, the DDR has a coronal inferomedial inclination (54). Because of such coronal inclination, a dural niche is created between the medial ICA and diaphragma sellae known as the carotid cave which is in the subarachnoid space (56).

Moving toward the base of the ACP, the upper dural covering of the ACP continues further medially as the falciform ligament, which covers 2–3 mm of the optic nerve proximal to the optic canal (Figure 6C) (56–58). Medially, this dural fold continues on the planum sphenoidale and merges posteriorly with the diaphragma sellae.

### Dural folds of the paraclinoid region

Inferior to DDR, the dural covering of the inferior surface of the ACP continues to descend on the ICA while encircling its lateral aspect. A few millimeters inferior to the DDR, this dural attachment thickens as the proximal dural ring (PDR). The PDR is basically the anterior continuation of the roof of the cavernous sinus below the ACP and separates the upper surface of the distal few millimeters of the oculomotor nerve from the ACP and adjacent clinoidal ICA just before it enters the SOF; hence, the PDR is also named as the carotid oculomotor membrane. DDR and PDR meet posteriorly near the tip of the ACP. Anteriorly, this dural fold continues on the inferior surface of the optic strut making the superomedial roof of the SOF. The PDR is less defined on the medial aspect of the ICA attaching to the carotid sulcus. The segment of the ICA between the PDR and DDR is the clinoidal segment (Figure 6).

#### Meningo-orbital band

The meningo-orbital band (MOB) (aka “frontotemporal dural fold”) is a fold of endosteal dura that exits through the lateral (non-neural) compartment of the SOF to continue as the periorbita (Figures 3, 6). Surgically, this band tethers the temporal dura to the SOF. Therefore, the temporal lobe dura could be detethered safely by incising this band and a pretemporal approach could be started to the lateral wall of the cavernous sinus by elevation of the outer meningeal layer of the lateral wall of the cavernous sinus to expose the inner meningeal layer (59). In doing this, the entire ACP is also exposed extradurally. Different techniques are described to cut the MOB safely and efficiently (60–63). Cutting the MOB and the endosteal dural layer at rotundum and ovale foramina allows full exposure of the lateral wall of the cavernous sinus and Meckel's cave up to the petrous ridge (59). The meningo-orbital artery (i.e., recurrent meningeal branch of the lacrimal artery) runs in the MOB and anastomoses with the branches of the anterior division of the MMA (64).

### Cavernous sinus

The cavernous sinus is situated in the medial-most region of the MCF proper (Figure 6). The cavernous sinus is formed between the meningeal and endosteal layers of the dura extending from the petroclival area posteriorly to the SOF anteriorly (59). The actual venous space of the sinus houses the cavernous ICA, its accompanying sympathetic plexus, and the abducens nerve. The meningeal layer of the dura forms the lateral, superior, posterior, and the upper (sellar) part of the medial walls. The periosteal layer forms the inferior wall, and the lower (sphenoidal) part of the medial wall. Upon entering the cavernous sinus, cranial nerves III, IV, and V1 are accompanied by a sleeve of meningeal layer. Therefore, the roof and the lateral wall are composed of a double meningeal layer of dura except for the area of the anterior clinoid process, which has a more complex anatomy (see above). When the MOB (i.e., the endosteal dural layer) is incised, the medial dura of the temporal lobe (i.e., the meningeal layer), which forms the true lateral wall of the cavernous sinus, could be peeled away from the meningeal layer that encases the individual nerves in the lateral wall. This inner membrane seals the venous space of the cavernous sinus laterally (59).

### Meckel's cave

Meckel's cave is a space between the two layers of the dura mater extending across the petrous apex that encircles the trigeminal root and ganglion, named after the German anatomist Johann Friedrich Meckel (1724–1774). The cave starts from porus trigeminus under the superior petrosal sinus where the cisternal segment of the trigeminal nerve crosses the petrous ridge above the trigeminal depression. The subarachnoid space extends beyond the ostium of Meckel's cave (i.e., trigeminal cistern). The point of distal cul de sac of Meckel's cave is a matter of controversy. A plausible understanding is that the subarachnoid space from the posterior cranial space usually extends up to a variable extent beyond the posterior edge of the Gasserian ganglion, whereas the dural covering extends to the anterior margin of the ganglion or even up to the exit foramina of the divisions (59).

The meningeal structure of the Meckel's cave is as follows. From lateral to medial the following layers are encountered: (1) the outer meningeal layer of the Meckel's cave, which is continuous with the superficial meningeal layer of the lateral wall of the cavernous sinus (i.e., temporal lobe dura propria); (2) the superficial inner layer of the meningeal dural sleeve around the trigeminal nerve; (3) the trigeminal nerve; (4) the deep inner layer of the meningeal dural sleeve; and (5) the periosteal dura of Meckel's cave (65). The periosteal dura covers the lacerum segment of the ICA (petro-lingual ligament) and continues on the surface of sphenoid bone as the medial wall of the cavernous sinus (59). When the periosteal dural layer is incised at the lateral aspect of the middle fossa foramina, the outer meningeal layer is elevated from the inner layer, which eventually continues as the epineurium of the nerve fibers (65). Technically, incising the endosteal layer at the foramina means that one has entered the space between the meningeal and endosteal layers which may contain venous lakes. As the dissection continues farther posteriorly and medially toward the trigeminal root, the petrous ridge is reached, which contains the superior petrosal sinus (65). Farther medially the two meningeal layers of the dura continue as the tentorium cerebelli, which is a reflection of the meningeal layer (Figure 6).

## Conclusion

The osseous and dural anatomy of the middle fossa are the foundations of understanding its detailed neurovascular anatomy. In order to safely navigate the middle fossa, the surgeon must know every detail of its anatomy. This work summarizes our current knowledge of the middle fossa bony and meningeal anatomy.
